# Association of Influenza Activity and Environmental Conditions With the Risk of Invasive Pneumococcal Disease

**DOI:** 10.1001/jamanetworkopen.2020.10167

**Published:** 2020-07-13

**Authors:** Isha Berry, Ashleigh R. Tuite, Angela Salomon, Steven Drews, Anthony D. Harris, Todd Hatchette, Caroline Johnson, Jeff Kwong, Jose Lojo, Allison McGeer, Leonard Mermel, Victoria Ng, David N. Fisman

**Affiliations:** 1 Dalla Lana School of Public Health, University of Toronto, Toronto, Ontario, Canada; 2 Canadian Blood Services, Ottawa, Ontario, Canada; 3 University of Alberta, Edmonton, Alberta, Canada; 4 University of Maryland School of Medicine, Baltimore; 5 Nova Scotia Health Authority, Halifax, Nova Scotia, Canada; 6 Dalhousie University, Halifax, Nova Scotia, Canada; 7 Philadelphia Department of Public Health, Philadelphia, Pennsylvania; 8 Warren Alpert School of Medicine of Brown University, Providence, Rhode Island; 9 Rhode Island Hospital, Providence; 10 Public Health Agency of Canada, Guelph, Ontario, Canada

## Abstract

**Importance:**

*Streptococcus pneumoniae* is the most commonly identified cause of bacterial pneumonia, and invasive pneumococcal disease (IPD) has a high case fatality rate. The wintertime coseasonality of influenza and IPD in temperate countries has suggested that pathogen-pathogen interaction or environmental conditions may contribute to IPD risk.

**Objectives:**

To evaluate the short-term associations of influenza activity and environmental exposures with IPD risk in temperate countries and to examine the generalizability of such associations across multiple jurisdictions.

**Design, Setting, and Participants:**

This case-crossover analysis of 19 566 individuals with IPD from 1998 to 2011 combined individual-level outcomes of IPD and population-level exposures. Participants lived in 12 jurisdictions in Canada (the province of Alberta and cities of Toronto, Vancouver, and Halifax), Australia (Perth, Sydney, Adelaide, Brisbane, and Melbourne), and the United States (Baltimore, Providence, and Philadelphia). Data were analyzed in 2019.

**Exposures:**

Influenza activity, mean temperature, absolute humidity, and UV radiation at delays of 1 to 3 weeks before case occurrence in each jurisdiction.

**Main Outcomes and Measures:**

Matched odds ratios (ORs) for IPD associated with changes in exposure variables, estimated using multivariable conditional logistic regression models. Heterogeneity in effects across jurisdictions were evaluated using random-effects meta-analytic models.

**Results:**

This study included 19 566 patients: 9629 from Australia (mean [SD] age, 42.8 [30.8] years; 5280 [54.8%] men), 8522 from Canada (only case date reported), and 1415 from the United States (only case date reported). In adjusted models, increased influenza activity was associated with increases in IPD risk 2 weeks later (adjusted OR [aOR] per SD increase, 1.07; 95% CI, 1.01-1.13). Increased humidity was associated with decreased IPD risk 1 week later (aOR per 1 g/m^3^, 0.98; 95% CI, 0.96-1.00). Other associations were heterogeneous; metaregression suggested that combinations of environmental factors might represent unique local risk signatures. For example, the heterogeneity in effects of UV radiation and humidity at a 2-week lag was partially explained by variation in temperature (UV index: coefficient, 0.0261; 95% CI, 0.0078 to 0.0444; absolute humidity: coefficient, −0.0077; 95% CI, −0.0125 to −0.0030).

**Conclusions and Relevance:**

In this study, influenza was associated with increased IPD risk in temperate countries. This association was not explained by coseasonality or case characteristics and appears generalizable. Absolute humidity was associated with decreased IPD risk in the same jurisdictions. The generalizable nature of these associations has important implications for influenza control and advances the understanding of the seasonality of this important disease.

## Key Points

**Question:**

What is the association of influenza activity and environmental conditions with invasive pneumococcal disease risk in temperate countries, and are these associations generalizable?

**Findings:**

In this case-crossover study of 19 566 patients from Australia, Canada, and the United States, influenza activity was associated with a short-term increase in risk of invasive pneumococcal disease, while absolute humidity was associated with a short-term decrease in invasive pneumococcal disease risk. These results were generalizable across the 3 temperate countries.

**Meaning:**

This study’s finding that influenza was associated with increased risk of invasive pneumococcal disease has important implications for disease control policy and practice.

## Teaser

This case-crossover study evaluates the short-term associations of influenza activity and environmental exposures with invasive pneumococcal disease risk in temperate countries and examines the generalizability of such associations across multiple jurisdictions.

## Introduction

Despite the availability of effective antimicrobial therapies and vaccines, invasive pneumococcal disease (IPD) remains an important cause of morbidity and mortality in high-, middle-, and low-income countries.^1,2,3,4^ More than 100 years after Sir William Osler characterized the pneumonia commonly caused by *Streptococcus pneumoniae* as the “captain of the men of death,”^5^ *S pneumoniae* remains the single most commonly recognized etiological agent in community-acquired pneumonia.^6^ Mortality from community-acquired pneumonia is generally high and may occur both in the presence and absence of invasive bacterial disease. However, invasive disease (including bacteremic pneumonia, meningitis, endocarditis, and septic arthritis) is associated with extremely high case fatality (11%-30% in North America and Asia, according to a 2014 review^7^). Pneumococcal conjugate vaccines have had a remarkable effect on reducing IPD,^8,9^ but replacement by virulent strains not included in available vaccine formulations has attenuated some of these gains.^10^ The emergence of penicillin-, macrolide-, and sulfa-resistant pneumococcal strains is also of concern.^11,12^

The seasonal cooccurrence of IPD and influenza has led to assertions of a causal relationship for more than 100 years. Osler noted in his text that Dr A. R. Reynolds, MD, the chief public health officer for the city of Chicago, linked the surge in deaths from bacterial pneumonia in that city to the reappearance of seasonal influenza epidemics after the 1889 influenza pandemic.^5^ However, Reynolds himself noted that the increase in pneumonia deaths had begun well before the 1889 pandemic and suggested that influenza was not solely responsible for increasing pneumonia incidence.^13^ The question of whether, and how, influenza and other respiratory viral infections may enhance the risk of subsequent pneumonia and invasive bacterial disease has been investigated by many groups, including our own.^14,15,16,17^ The question is important for disease control policy and practice: if influenza is indeed a causal factor in pneumonia and IPD, then prevention of IPD becomes an important and underappreciated benefit of seasonal influenza programs.

In prior work on IPD and influenza coseasonality in Toronto, Canada,^14^ we noted an apparent lack of correlation in the phase, amplitude, or peak timing of annual influenza and IPD waves but did find that increases in IPD risk occurred with a 1-week delay after increases in influenza activity. We concluded that enhanced risk of IPD associated with influenza occurred as a result of increased invasion among individuals with infection rather than increased risk of colonization (which should result in correlation of seasonal waveforms). This previous study used a self-matched design, so it was not subject to confounding by coseasonality or patient characteristics. However, because it was performed in a single center, the generalizability of this association might be questioned. Furthermore, while it adjusted for mean temperature, relative humidity, and UV radiation exposure, it did not adjust for confounding by absolute humidity, which has been shown to be an important predictor for influenza activity.^18^

To evaluate the generalizability of the association of influenza with IPD in a manner that adjusted for absolute humidity, we undertook to expand our earlier work to include multiple jurisdictions using identical methods. Our objectives were to evaluate the short-term associations of influenza activity and environmental exposures with IPD risk within each jurisdiction and to evaluate the generalizability of such associations across multiple jurisdictions.

## Methods

This is a multisite, multiyear, semi-ecological study that combined individual-level outcomes of IPD and population-level exposures of influenza and environmental factors. We obtained data from 12 jurisdictions across 3 countries, as follows: Australia (Adelaide, Brisbane, Melbourne, Perth, and Sydney), Canada (the province of Alberta and cities of Halifax, Toronto, and Vancouver), and the eastern United States (Baltimore, Philadelphia, and Providence) (eFigure 1 in the Supplement) from 1998 to 2011. Data from Alberta were only available at the provincial level, so we assigned these data a location at the midpoint between the province’s 2 major population centers (Edmonton and Calgary), which together account for more than half of the provincial population.^19^ Ethical approval for this study was obtained from the University of Toronto Research Ethics Board. This study used deidentified case data obtained from preexisting surveillance databases; thus, informed consent was not required. This study follows the Strengthening the Reporting of Observational Studies in Epidemiology (STROBE) reporting guideline for case-control studies.

We obtained IPD case data retrospectively from population-based or hospital-based surveillance systems operating in each of the included jurisdictions. All included jurisdictions considered IPD a notifiable disease, and a uniform case definition was used to ensure comparability across study sites. Invasive pneumococcal infection was defined as positive culture isolation of *S pneumoniae* from a normally sterile body site (usually blood, cerebrospinal fluid, or synovial fluid). Participating centers provided results of all cases with IPD-positive results, identified only by case date (ie, culture collection date), from 1998 to 2011. However, not all regions had case information for the entire date range. A full description of the IPD population-based and hospital-based data sources and available periods is summarized in eTable 1 in the Supplement.

Influenza data were also obtained from population-based surveillance systems and/or public health departments from all participating centers. Given that many jurisdictions reported influenza based on weekly counts, counts from jurisdictions reporting daily were aggregated to the weekly level. Influenza A and B counts were combined, and influenza was treated as an ecological (ie, ubiquitous) exposure for the jurisdictional population. Given that there are varying surveillance systems for capturing influenza among our regions (principally hospital-based reporting vs laboratory-based surveillance), raw influenza case counts were noncomparable across regions. Therefore, case counts were standardized by subtracting the mean and dividing counts by the standard deviation of the influenza time series for that jurisdiction. For each jurisdiction in our analyses, we interpreted associations with IPD as the relative risk per standard deviation change from the mean influenza count.

Daily meteorological data, including mean temperature, mean relative humidity, and maximum UV index (a composite measure of the erythrogenic effect of ambient UV radiation^20^), for the corresponding study period for each region were obtained through federal weather and atmospheric agencies. Our choice of these exposures reflects both the availability of measurements and their identification as variables associated with the seasonality of both influenza and invasive bacterial respiratory disease.^17,18,20,21,22^ In each region, we obtained data from the weather station of the largest airport serving the area. If airport weather stations were unavailable or were missing data, we used the next closest station. If there were multiple meteorological readings per a day, we calculated daily means. Measures of relative humidity were converted to absolute humidity (AH; in grams per meter cubed) as a function of mean temperature (T_mean_) and relative humidity (RH) using the heuristic AH = 6.11 × RH% × 10^(7.5 × Tmean/[237.7 + Tmean])^. For all analyses, we averaged the data temporally and spatially to create weekly time series of environmental conditions for each jurisdiction. Data sources for meteorological and infectious disease covariates are presented in eTable 1 in the Supplement.

### Statistical Analysis

The monthly proportion of IPD cases was examined graphically to evaluate seasonality; seasonal oscillation was evaluated statistically using Poisson regression models with fast Fourier transforms. Evidence of seasonal oscillation in each jurisdiction was defined as *P* < .05 for combined sine and cosine terms in the models.

A time-stratified 2:1 matched case-crossover design was then used to evaluate short-term associations of influenza and environmental factors with IPD risk in each region. A case-crossover design can be thought of as a type of self-matched case-control study, in which each case serves as its own control.^23,24^ In our study, a case was defined as a day on which an IPD case occurred, and controls were defined as temporally proximate days on which a case did not occur. Beginning 3 weeks before the first IPD case date reported in each region, the total person-time at risk was divided into 3-week time strata. Controls were chosen by matching the day of the week to the case within each 3-week stratum and could both precede, both follow, or straddle the case day. Therefore, each stratum consisted of 1 case day and 2 control days for the same individual (eFigure 2 in the Supplement). Random directionality of control selection was used to avoid biases from seasonal patterns that can occur with unidirectional or uniform bidirectional control selection.^25^ We evaluated the associations of influenza exposures and environmental covariates, allowing for potential lag effects of as long as 3 weeks. Within each region, crude odds ratios (ORs) were calculated for IPD by each exposure and lag period (ie, 4 exposures × 3 lag periods, resulting in 12 covariates) using conditional logistic regression models. Multivariable models were constructed for each region to obtain region-specific adjusted ORs (aORs) with 95% CIs. This study design automatically controls for time-invariant individual-level confounders, given that comparisons are made within individuals.

Following this, we estimated the pooled effect for each covariate using a random-effects meta-analytic model. Between-region homogeneity and heterogeneity of effects were identified using the meta-analytic Cochran Q statistic.^26^ If there was some evidence of heterogeneity (ie, *P* < .10), we constructed metaregression models to identify and explore sources of heterogeneity. Univariable and multivariable metaregression models were constructed using region-specific characteristics as explanatory variables, including mean and variation in environmental exposures, population density, and distance from the equator (modeled as latitude squared). All analyses were conducted in Stata version 13.0 (StataCorp). Statistical significance was set at *P* < .05, and all tests were 2-tailed.

## Results

**Figure 1.**
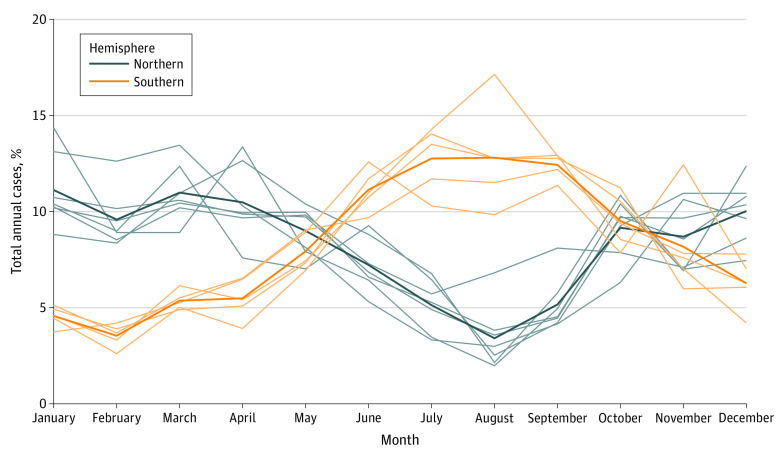
Seasonality of Invasive Pneumococcal Disease by Hemisphere Bold curves represent hemisphere means and demonstrate inversion of seasonal waves. Individual jurisdictions are labeled in eFigure 3 in the Supplement.

A total of 19 566 IPD cases occurred during the study period with influenza and environmental exposure data available: 9629 from Australia (mean [SD] age, 42.8 [30.8] years; 5280 [54.8%] men), 8522 from Canada (only case date reported), and 1415 from the United States (only case date reported) (Table 1). Owing to data restrictions, demographic data was not available for analysis in Canada and the United States. In all regions, Fourier transforms in seasonal regression models identified seasonality of IPD risk. We observed that IPD displayed wintertime predominance, with the seasonal peak in January to April in the northern hemisphere (Canada and the United States) and in June to September in the southern hemisphere (Australia) (Figure 1; eFigure 3 in the Supplement). For example, we found that January cases accounted for 11.1% of all cases in cities in the northern hemisphere, but only 4.6% in Australian cities. By contrast, 12.8% of cases occurred in July in Australia, while only 5.1% of cases in northern cities occurred in this month.

**Table 1.** Number of IPD Cases and aORs for IPD With Lagged Exposure to Influenza, 1998 to 2011

Jurisdiction	IPD Cases, No.	aOR (95% CI), by lag^a^		
		1 wk	2 wk	3 wk
Australia	9629	NA	NA	NA
Adelaide	1206	0.79 (0.49-1.27)	1.14 (0.59-2.21)	1.16 (0.78-1.75)
Brisbane	1738	1.07 (0.87-1.31)	1.01 (0.76-1.34)	1.11 (0.91-1.36)
Melbourne	2568	1.02 (0.86-1.22)	0.92 (0.74-1.15)	1.06 (0.92-1.24)
Perth	1173	0.74 (0.53-1.05)	1.28 (0.77-2.12)	1.19 (0.89-1.60)
Sydney	2944	0.96 (0.83-1.12)	1.18 (1.01-1.38)	0.98 (0.85-1.13)
Canada	8522	NA	NA	NA
Alberta	527	0.89 (0.70-1.13)	0.74 (0.34-1.60)	1.51 (0.65-3.51)
Halifax	297	1.11 (0.71-1.74)	1.00 (0.61-1.62)	1.57 (1.07-2.31)
Toronto	4822	1.07 (0.97-1.18)	1.05 (0.92-1.19)	0.91 (0.82-1.02)
Vancouver	2876	1.03 (0.95-1.13)	1.08 (0.99-1.16)	1.07 (0.97-1.18)
United States	1415	NA	NA	NA
Baltimore	659	1.07 (0.86-1.33)	1.03 (0.79-1.34)	0.91 (0.71-1.16)
Philadelphia	558	1.12 (0.74-1.70)	1.36 (0.84-2.21)	0.88 (0.56-1.37)
Providence	198	1.19 (0.81-1.74)	0.57 (0.24-1.36)	1.49 (0.70-3.17)

Abbreviations: aOR, adjusted odds ratio; IPD, invasive pneumococcal disease; NA, not applicable.

^a^ Odds ratios were generated using conditional logistic regression, adjusting for standardized influenza, mean temperature, absolute humidity, and UV index at lags of 1 to 3 weeks.

Region-specific multivariable models included standardized influenza rate, temperature, absolute humidity, and UV exposures at lags of 1 to 3 weeks. In these models, influenza was associated with a significant increase in IPD risk with a 2-week lag in Sydney (aOR, 1.18; 95% CI, 1.01-1.38) and a 3-week lag in Halifax (aOR, 1.57; 95% CI, 1.07-2.31) (Table 1). Associations between IPD and environmental exposures were similarly variable (eTable 2 in the Supplement). However, when effects were pooled across jurisdictions, we found homogeneous increases in IPD risk associated with increased influenza activity at lags of 1, 2, and 3 weeks; this association was statistically significant for influenza activity at a 2-week lag (pooled OR for each SD increase in influenza, 1.07; 95% CI, 1.01-1.13; *I*^2^ = 0%; *P* = .02) (Table 2).^26,27^ Absolute humidity at a 1-week lag was significantly and homogeneously associated with reduced risk of IPD (pooled OR per 1 g/m^3^, 0.98; 95% CI, 0.96-1.00; *I*^2^ = 0%; *P* = .02) (Table 2).

**Table 2.** Pooled Effects Between Invasive Pneumococcal Disease, Influenza, and Environmental Factors Across 12 Regions, 1998 to 2011

Variable, by lag	Pooled OR (95% CI)^a^	I^2^ statistic, %^b^	*P* value for heterogeneity^c^
Influenza			
1 wk	1.03 (0.98-1.08)	0.00	.69
2 wk	1.07 (1.01-1.13)	0.00	.70
3 wk	1.04 (0.97-1.12)	30.4	.15
Temperature			
1 wk	1.01 (0.99-1.03)	56.4	.01
2 wk	0.99 (0.97-1.01)	42.7	.06
3 wk	0.99 (0.97-1.01)	48.2	.03
UV index			
1 wk	0.96 (0.92-1.01)	39.5	.08
2 wk	0.99 (0.93-1.06)	64.7	.001
3 wk	1.00 (0.95-1.05)	45.2	.04
Absolute humidity			
1 wk	0.98 (0.96-1.00)	0.00	.75
2 wk	0.99 (0.96-1.02)	57.2	.01
3 wk	0.98 (0.96-1.01)	50.4	.02

Abbreviation: OR, odds ratio.

^a^ Pooled ORs were calculated using random-effects meta-analytic model of 12 jurisdiction-specific adjusted ORs. Jurisdiction-specific models were adjusted for influenza, temperature, absolute humidity, and UV index at lags of 1 to 3 weeks.

^b^ The *I*^2^ statistic may be interpreted as the proportion of variation that is attributable to between-model heterogeneity rather than within-model variability.^27^

^c^ *P *value based on Cochran Q statistic.^26^

**Figure 2.**
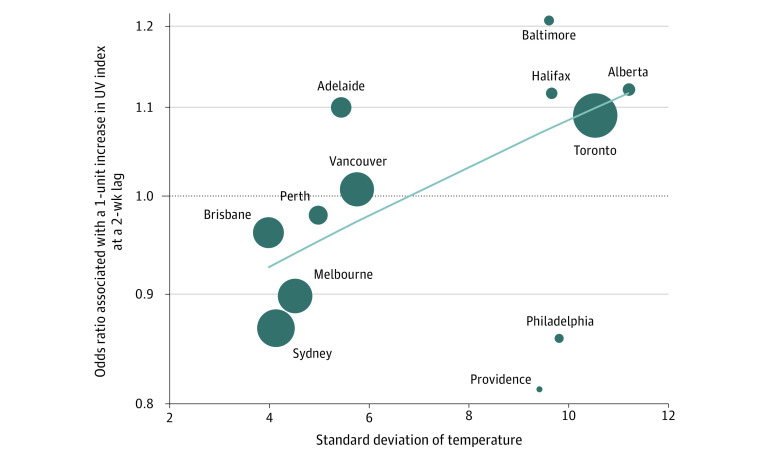
Interaction Between UV Radiation Effects and Standard Deviation of Local Temperature Marker size is proportional to inverse of variance. The solid line represents a weighted regression line obtained via metaregression, and the dashed line at 1.0 indicates an odds ratio of 1.0, the equivalent of no effect.

Other pooled environmental exposures with a 1-week lag were associated with heterogeneity of effect (pooled OR for 1-°C increase in temperature, 1.01; 95% CI, 0.99 to 1.03; *I*^2^ = 56.4%; pooled OR for 1-unit increase in UV index, 0.96; 95% CI, 0.92 to 1.01; *I*^2^ = 39.5%). In exploratory metaregression models, we found that heterogeneity in the effects of temperature at all lags was explained partially by variability in other exposures (ie, standard deviations of humidity and influenza exposures), while the heterogeneity in effects of UV radiation and humidity at a 2-week lag was partially explained by variation in temperature (UV index: coefficient, 0.0261; 95% CI, 0.0078 to 0.0444; absolute humidity: coefficient, −0.0077; 95% CI, −0.0125 to −0.0030) (Table 3). Thus, the associations of some exposures with IPD risk appeared to be modified by underlying climatic conditions in a given jurisdiction. The variability in the effect of UV radiation on risk, according to standard deviation of temperature in jurisdictions, is shown in Figure 2. In jurisdictions with less temperature variability, UV radiation was associated with decreased IPD risk, while UV radiation was associated with an increase in downstream IPD where there was more variation in temperature. Other metaregression results are illustrated graphically in eFigure 4 to eFigure 7 in the Supplement.

**Table 3.** Coefficients From Metaregression Models

Lag, wk	Exposure	Explanatory variable	Coefficient (95% CI)^a^
1	Temperature	SD of absolute humidity	0.0169 (0.0021 to 0.0317)
2	Temperature	SD of influenza	–0.0030 (–0.0058 to –0.0001)
2	UV index	SD of temperature	0.0261 (0.0078 to 0.0444)
2	Absolute humidity	Mean temperature	–0.0077 (–0.0125 to –0.0030)
3	Temperature	SD of absolute humidity	0.0118 (0.0003 to 0.0234)

^a^ The coefficient may be interpreted as estimated change in log(odds ratio) of exposure for each unit change in the explanatory variable.

## Discussion

We evaluated the association of influenza and environmental exposures (temperature, absolute humidity, and UV radiation exposure) with IPD risk in multiple jurisdictions in high-income, temperate countries (Canada, the United States, and Australia). Using a case-crossover approach that implicitly controls for seasonal covariation in disease risk, we found that increases in influenza activity were associated with increases in IPD risk 2 weeks later and that this association appeared homogeneous regardless of the country or hemisphere in which it was evaluated. We also found that increased absolute humidity was associated with decreased IPD risk at a lag of 1 week and that this association was similarly homogeneous across jurisdictions. Lastly, and intriguingly, we found that other environmental exposures were also associated with IPD risk but that these associations appeared specific to a given jurisdiction, with evidence from metaregression models suggesting that some of this variability was determined by average environmental conditions or variability in environmental conditions in each jurisdiction.

Our finding that influenza was associated with increased risk of IPD during subsequent weeks affirms our earlier observations, which were restricted to the Toronto area in Canada,^14^ but suggests that this association is generalizable, at least in temperate countries like those included in the present study. If correct, this has important implications for vaccine policy and suggests that prevention of invasive bacterial disease may be an important potential benefit of influenza prevention programs.^28^ While we have not attempted attributable risk calculations in the current analysis, methods for such estimation are straightforward and would allow this benefit to be quantified.^29,30,31^ The mechanisms whereby upstream viral infection might predispose those with the infection to invasive bacterial infection might include exposure of pneumococcal binding sites by viral neuraminidase^15,32^; decreased bacterial clearance, mediated by interferon gamma release during clearance of viral infection^33^; and depletion of alveolar macrophages by influenza infection.^34^ We have previously identified associations of influenza with subsequent risks of invasive meningococcal disease,^35^ and the association between influenza and subsequent necrotizing *S. aureus* pneumonia is well described,^36^ raising the question of whether enhanced risk of IPD after influenza reflects enhanced risk of invasive bacterial respiratory disease more generally.

An unexpected finding in this analysis was that the effect estimate for absolute humidity (ie, the absolute quantity of water per unit volume of atmosphere) was in the protective direction and that this was independent of influenza activity. Our reason for including absolute humidity in these analyses was the finding by Shaman et al^18^ that higher humidity attenuates the risk of influenza season onset, which in turn is consistent with experimental data suggesting greater influenza transmissibility in drier conditions.^37,38^ However, the association that we described here cannot be mediated via influenza, because it occurs at a shorter lag than that seen with influenza. It is plausible that drier air may increase the propensity of *S pneumoniae* to invade because of increased mucus viscosity or diminished ciliary function, effects well described with dry air ventilation in the anesthetic literature.^39,40^

For many of the environmental exposures we evaluated, heterogeneity in effects was seen across regions. However, exploratory metaregression models suggested that this variation may be because of interactions between short-term environmental exposures and underlying climatic conditions in a given region. The possibility that unique environmental risk-signatures exist for environmental influence on disease in different regions is intriguing and may suggest a degree of pathogen adaptation to varying environmental conditions over time. It may also explain the inconsistency in reported environmental influences on IPD risk reported in prior studies.^20,22,41,42^

### Limitations

This study has limitations. We included large numbers of covariates in our conditional logistic regression models to use a uniform approach across jurisdictions. This likely resulted in variance-bias trade-off,^43^ which explains why we did not find associations between influenza and IPD risk in many of our individual regions but did see this risk when variance was reduced again via meta-analysis. Like any ecological study, exposure status may be misclassified (eg, if cases were in a different locale during the control period). Given that we based our study on reported cases of invasive bacterial disease, cases were likely to be undercounted. However, case counting is unlikely to be associated with exposure measures and so is unlikely to have introduced bias; random misclassification would have introduced bias toward the null, so the associations we observed likely represent lower bounds. Furthermore, we did not include particulate air pollution as an exposure in this study, restricting our environmental covariates to meteorological exposures. Air pollution may also enhance the risk of IPD,^20,44,45^ and this association deserves further exploration.

## Conclusions

The question of whether influenza is associated with IPD incidence is of considerable public health importance, both with respect to attribution of risk and for the development of preventive strategies. By applying a consistent, self-matched approach across multiple jurisdictions, the findings of this study demonstrated a consistent association between short-term increases in influenza and subsequent increases in IPD. The methods used in this study make it unlikely that the findings were because of confounding by factors associated with the wintertime seasonality of disease and are independent of environmental factors (which themselves are associated with IPD risk). We suggest that the estimation of attributable fractions of IPD because of influenza could result in a reassessment of the cost-effectiveness of influenza vaccination programs.

## Notes

Supplement. **eFigure 1. **Map of Included Jurisdictions **eFigure 2. **Schematic Diagram of Control Selection Scheme with Random Directionality **eFigure 3. **Seasonality of Invasive Pneumococcal Disease by City **eFigure 4. **Interaction Between Absolute Humidity and Mean Local Temperature **eFigure 5. **Interaction Between Temperature and Variability in Absolute Humidity With 1-Week Lag **eFigure 6. **Interaction Between Temperature and Variability in Absolute Humidity With 3-Week Lag **eFigure 7. **Interaction Between Temperature and Variability in Influenza Activity **eTable 1. **Jurisdiction, Time-Period, and Data Sources for Infectious Disease and Environmental Data **eTable 2. **Adjusted Odds Ratios for IPD With Lagged Exposure to Absolute Humidity, Temperature, or UV Radiation, 1998 to 2011
