# Supplementary material for: Association of Influenza Activity and Environmental Conditions With the Risk of Invasive Pneumococcal Disease
Source: JAMA Netw Open. 2020 Jul 13;3(7):e2010167. doi: 10.1001/jamanetworkopen.2020.10167 (PMC7358913; doi:10.1001/jamanetworkopen.2020.10167)
Supplement: Supplement. — eFigure 1. Map of Included Jurisdictions eFigure 2. Schematic Diagram of Control Selection Scheme with Random Directionality eFigure 3. Seasonality of Invasive Pneumococcal Disease by City eFigure 4. Interaction Between Absolute Humidity and Mean Local Temperature eFigure 5. Interaction Between Temperature and Variability in Absolute Humidity With 1-Week Lag eFigure 6. Interaction Between Temperature and Variability in Absolute Humidity With 3-Week Lag eFigure 7. Interaction Between Temperature and Variability in Influenza Activity eTable 1. Jurisdiction, Time-Period, and Data Sources for Infectious Disease and Environmental Data eTable 2. Adjusted Odds Ratios for IPD With Lagged Exposure to Absolute Humidity, Temperature, or UV Radiation, 1998 to 2011 [file jamanetwopen-3-e2010167-s001.pdf]

Supplementary Online Content

Berry I, Tuite AR, Salomon A, et al. Association of influenza activity and environmental conditions with the risk of invasive pneumococcal disease. *JAMA Netw Open*. 2020;3(7):e2010167. doi:10.1001/jamanetworkopen.2020.10167

eFigure 1. Map of Included Jurisdictions
eFigure 2. Schematic Diagram of Control Selection Scheme with Random Directionality
eFigure 3. Seasonality of Invasive Pneumococcal Disease by City
eFigure 4. Interaction Between Absolute Humidity and Mean Local Temperature
eFigure 5. Interaction Between Temperature and Variability in Absolute Humidity With 1-Week Lag
eFigure 6. Interaction Between Temperature and Variability in Absolute Humidity With 3-Week Lag
eFigure 7. Interaction Between Temperature and Variability in Influenza Activity
eTable 1. Jurisdiction, Time-Period, and Data Sources for Infectious Disease and Environmental Data
eTable 2. Adjusted Odds Ratios for IPD With Lagged Exposure to Absolute Humidity, Temperature, or UV Radiation, 1998 to 2011

This supplementary material has been provided by the authors to give readers additional information about their work.

© 2020 Berry I et al. *JAMA Network Open*.

eFigure 1. Map of Included Jurisdictions

Green dots represent cities from which either population- or hospital-based case counts were derived. Polygon represents the Canadian Province of Alberta; the midpoint between the cities of Edmonton and Calgary was used for analytic purposes.

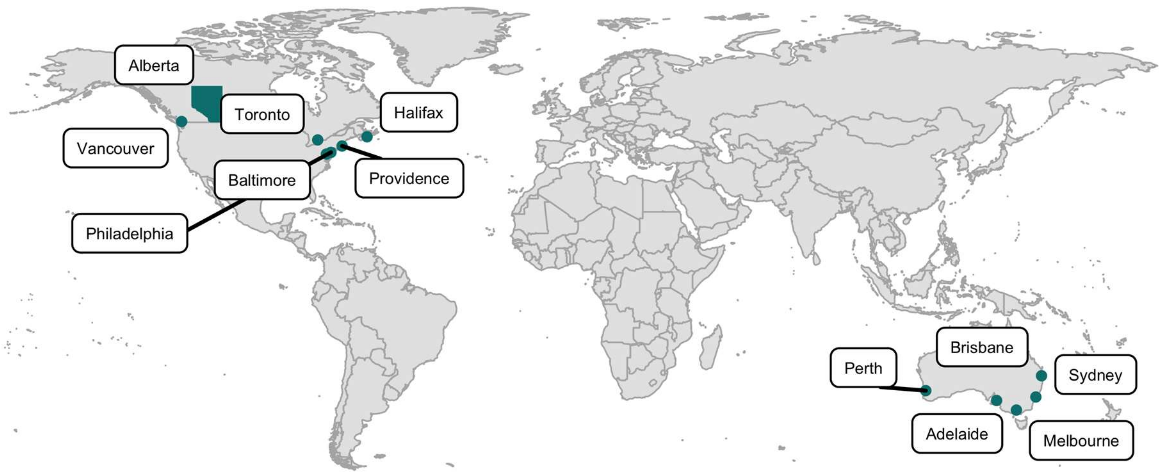

© 2020 Berry I et al. *JAMA Network Open*.

eFigure 2. Schematic Diagram of Control Selection Scheme with Random Directionality

Schematic diagram of control selection strategy for case-crossover study. Each row represents a 3-week time block. Hazard and control periods (matched by day-of-week) are selected from the 3-week time block, resulting in random directionality of control selection.

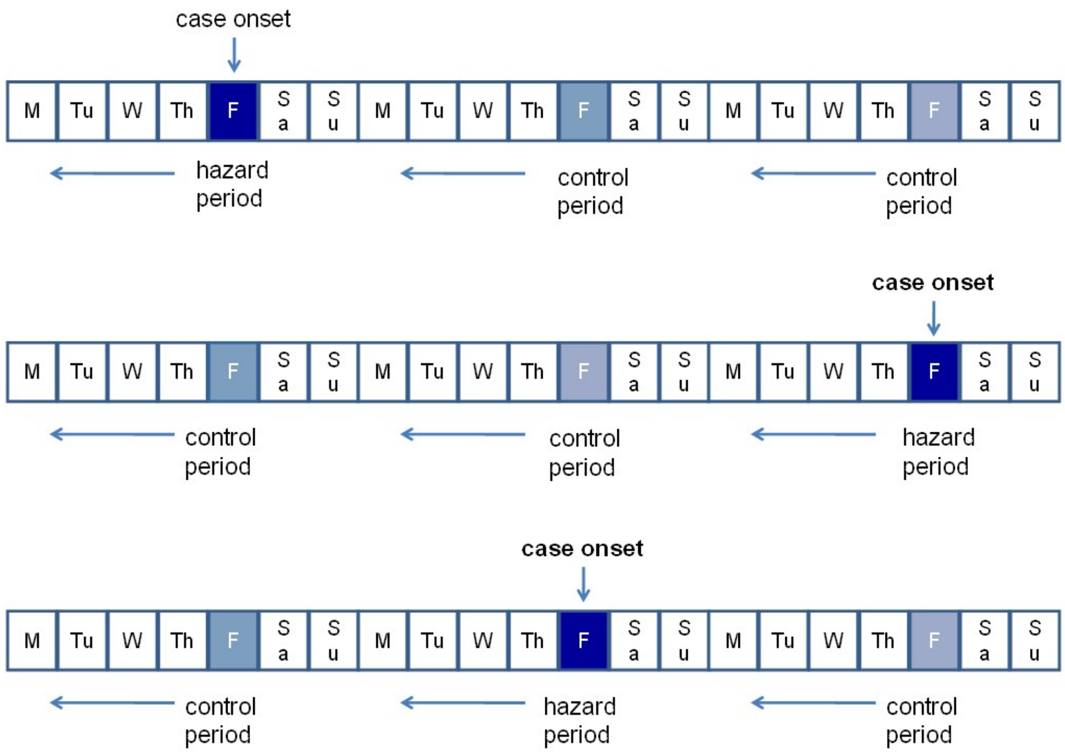

© 2020 Berry I et al. *JAMA Network Open.*

eFigure 3. Seasonality of Invasive Pneumococcal Disease by City

Percentage of invasive pneumococcal cases (Y-axis) are plotted by month (X-axis) for northern hemisphere (top panel A) and southern hemisphere (bottom panel B) jurisdictions. Legend identifies individual jurisdictions.

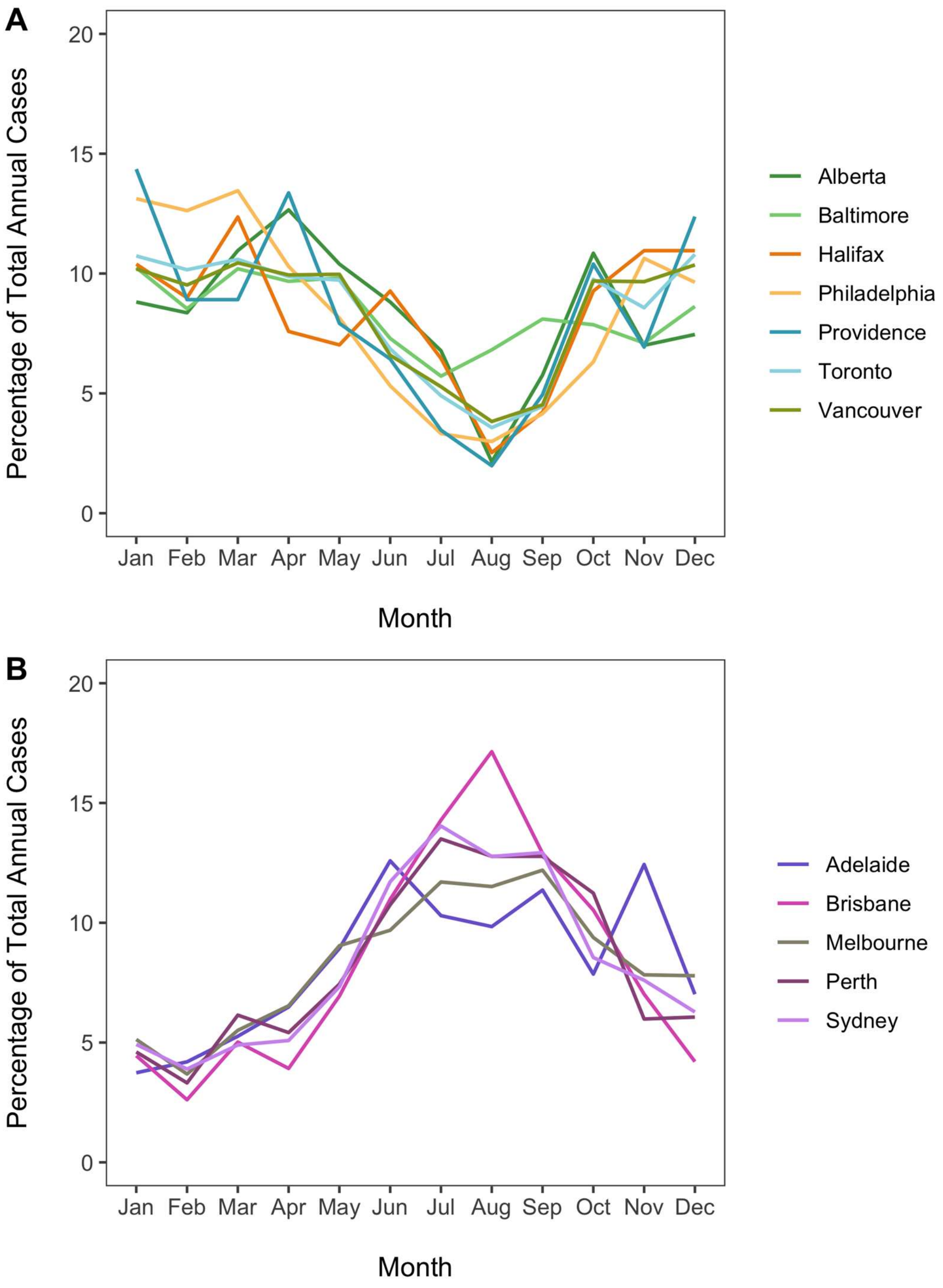

© 2020 Berry I et al. *JAMA Network Open.*

eFigure 4. Interaction Between Absolute Humidity and Mean Local Temperature

The odds ratio associated with a 1 g/m^3 increase in absolute humidity at a 2-week lag (Y-axis) is plotted against mean temperature (°C) (X-axis). Marker size is proportional to inverse of variance. The solid line represents a weighted regression line obtained via meta-regression. In colder jurisdictions, absolute humidity appears to be associated with an increase in downstream IPD risk, while absolute humidity is protective against IPD in warmer jurisdictions.

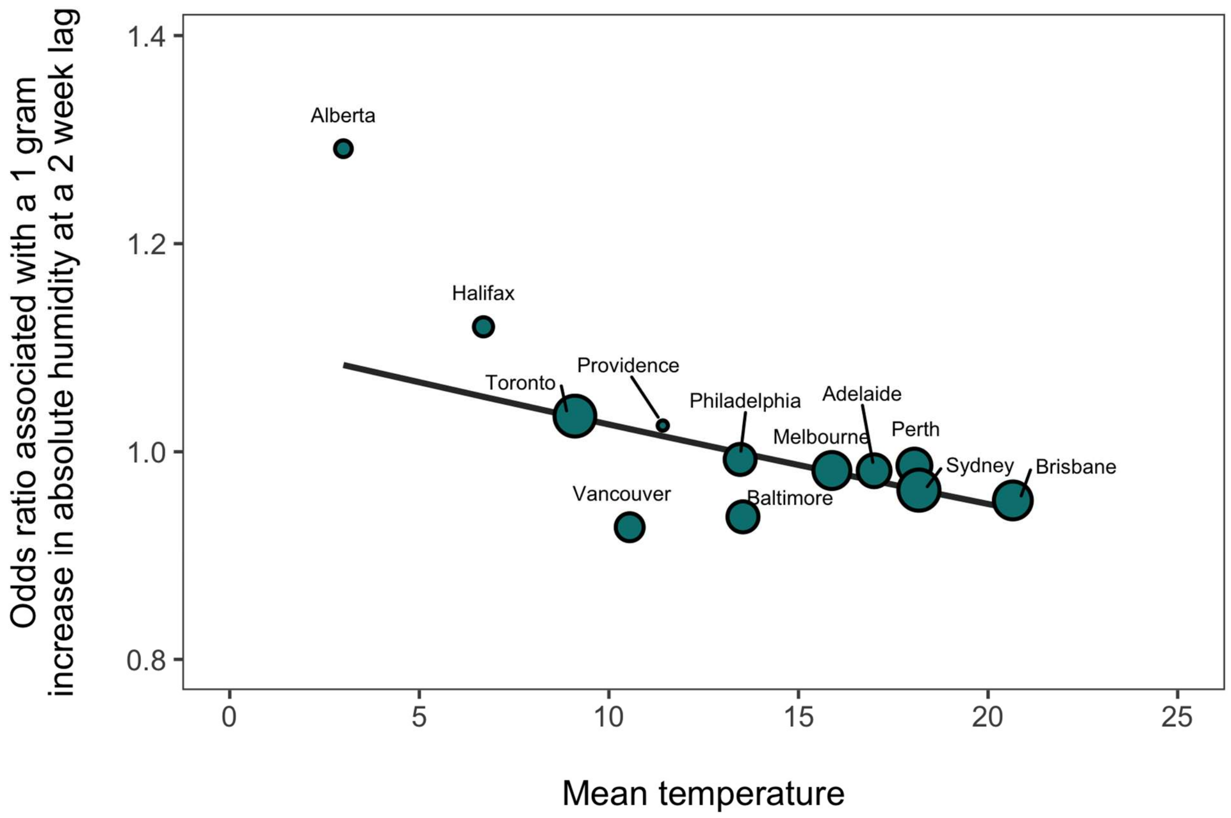

© 2020 Berry I et al. *JAMA Network Open.*

eFigure 5. Interaction Between Temperature and Variability in Absolute Humidity With 1-Week Lag

The odds ratio associated with a 1°C increase in temperature at a 1-week lag (Y-axis) is plotted against local variability of absolute humidity (X-axis). Marker size is proportional to inverse of variance. The solid line represents a weighted regression line obtained via meta-regression. In regions with less variability in humidity, increasing temperature appears to be associated with a decrease in downstream IPD risk, while increasing temperature is associated with increased risk in regions where humidity is more variable.

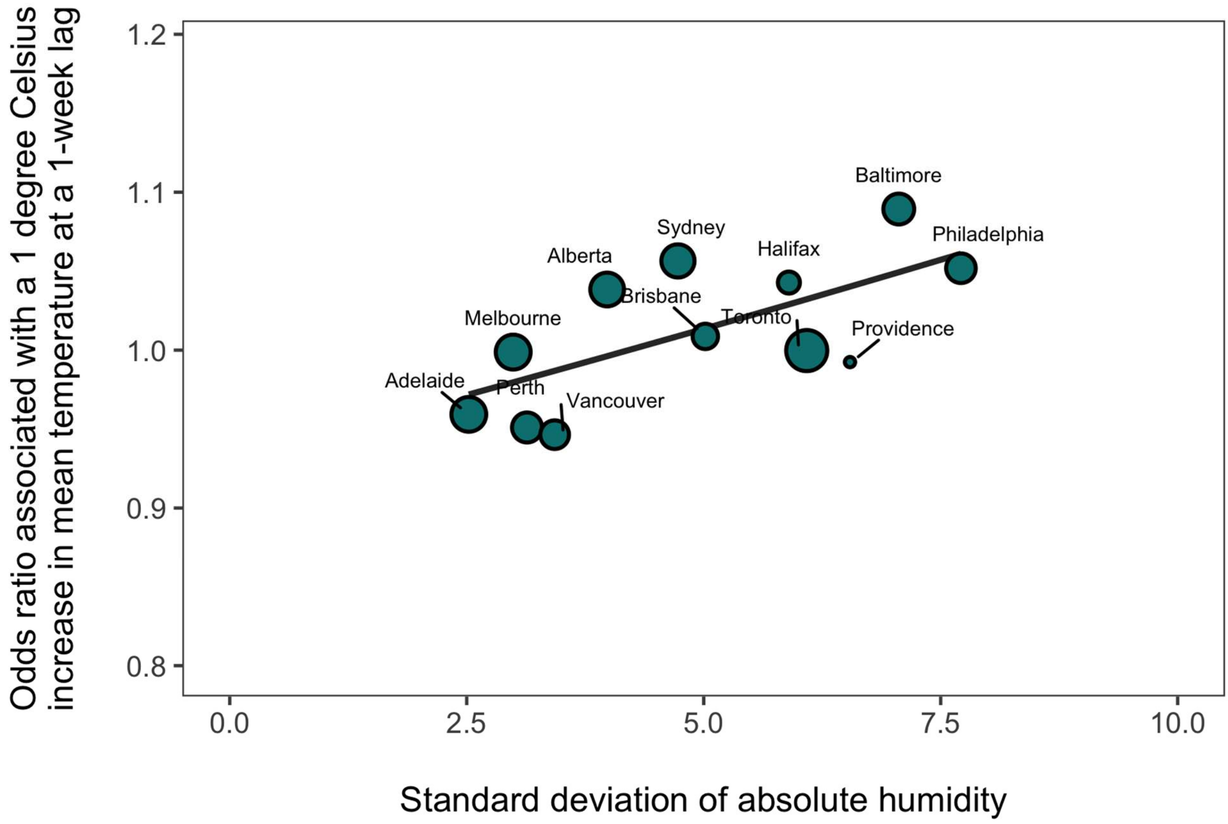

© 2020 Berry I et al. *JAMA Network Open.*

eFigure 6. Interaction Between Temperature and Variability in Absolute Humidity With 3-Week Lag

The odds ratio associated with a 1°C increase in temperature at a 3-week lag (Y-axis) is plotted against local variability of absolute humidity (X-axis). Marker size is proportional to inverse of variance. The solid line represents a weighted regression line obtained via meta-regression. In regions with less variability in humidity, increasing temperature appears to be associated with a decrease in downstream IPD risk, while increasing temperature is associated with increased risk in regions where humidity is more variable. Effect is similar to that demonstrated in Supplementary Figure 3 but occurs at a longer lag.

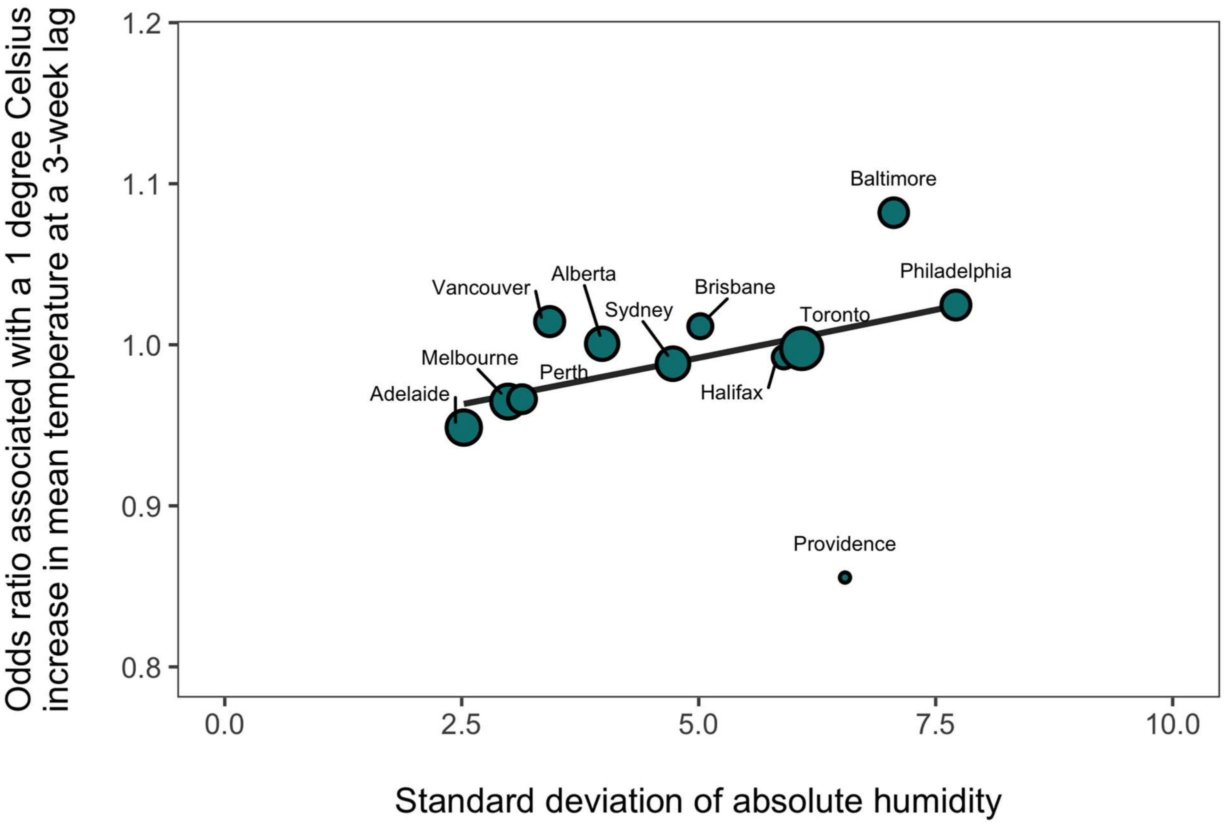

© 2020 Berry I et al. *JAMA Network Open.*

eFigure 7. Interaction Between Temperature and Variability in Influenza Activity

The odds ratio associated with a 1°C increase in temperature at a 2-week lag (Y-axis) is plotted against local variability in influenza activity (X-axis). Marker size is proportional to inverse of variance. The solid line represents a weighted regression line obtained via meta-regression. In regions with less variability in influenza activity, increasing temperature appears to be associated with an increase in downstream IPD risk, while increasing temperature is associated with decreased risk in regions where influenza activity is more variable.

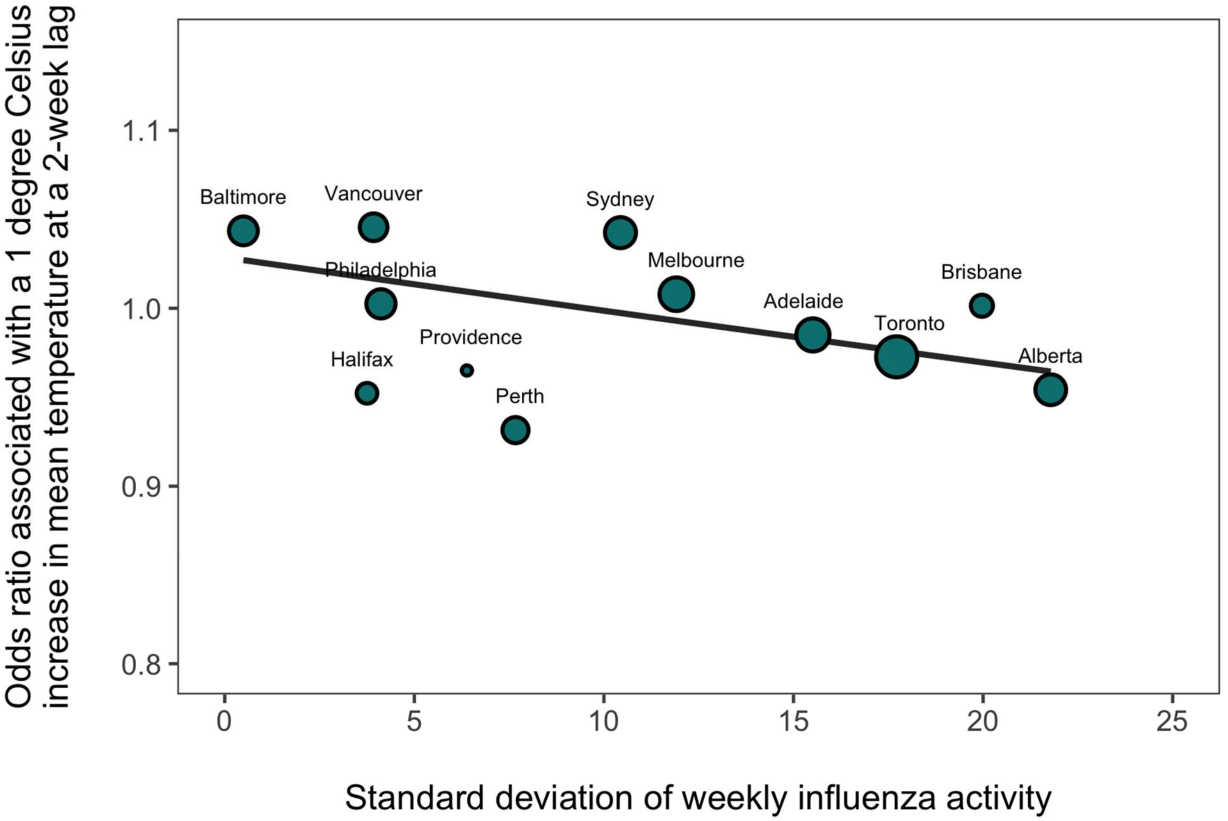

© 2020 Berry I et al. *JAMA Network Open.*

eTable 1. Jurisdiction, Time-Period, and Data Sources for Infectious Disease and Environmental Data

Country	Region	Start Date	End Date	Invasive Pneumococcal Disease	Influenza	Temperature	Humidity	Ultraviolet Index
Australia	Adelaide	Apr 2001	Nov 2011	Australian National Notifiable Disease Surveillance System (NNDSS)	Australian National Notifiable Disease Surveillance System (NNDSS)	Australian Government, Bureau of Meteorology (BOM)	Australian Government, Bureau of Meteorology (BOM)	Australian Radiation Protection and Nuclear Safety Agency (ARPANSA)
	Brisbane	Jul 2001	Dec 2011					
	Melbourne	Jul 2001	Dec 2011					
	Perth	Dec 2000	Dec 2011					
	Sydney	Jan 2001	Dec 2011					
Canada	Alberta (modeled as midpoint between Calgary & Edmonton)	Jan 2005	Oct 2009	Alberta Provincial Public Health Laboratory	Public Health Agency of Canada (FluWatch)	Environment Canada	Environment Canada	World Ozone and Ultraviolet Radiation Data Centre (WOUDC)
	Halifax	Jan 2001	Oct 2009	Queen Elizabeth II Health Science Centre				
	Toronto	Jan 1998	Oct 2009	Toronto Invasive Bacterial Diseases Network (TIBDN)				
	Vancouver	Feb 2000	Oct 2009	British Columbia Centre for Disease Control				
USA	Baltimore	Sept 2007	Dec 2011	University of Maryland Medical Center	University of Maryland Medical Center	National Oceanic and Atmospheric Administration (NOAA) Climate Prediction Centre	National Oceanic and Atmospheric Administration (NOAA) Climate Prediction Centre	National Oceanic and Atmospheric Administration (NOAA) Climate Prediction Centre
	Philadelphia	Jan 2002	Apr 2007	Philadelphia Department of Public Health	Philadelphia Department of Public Health			
	Providence	Jun 2005	Jul 2011	Rhode Island Hospital	Rhode Island Hospital			

© 2020 Berry I et al. *JAMA Network Open.*

eTable 2. Adjusted Odds Ratios for IPD With Lagged Exposure to Absolute Humidity, Temperature, or UV Radiation, 1998 to 2011

Jurisdiction	Adjusted Odds Ratio (by Lag) (95% CI)								
	Mean Temperature (°C)			Absolute Humidity (g/m³)			UV Index		
	1-week	2-week	3-week	1-week	2-week	3-week	1-week	2-week	3-week
**Australia**									
Adelaide	0.96 (0.94, 1.00)	0.98 (0.96, 1.03)	0.95 (0.93, 0.99)	0.98 (0.91, 1.05)	0.98 (0.95, 1.06)	1.01 (0.97, 1.08)	0.91 (0.85, 1.04)	1.09 (1.02, 1.27)	1.12 (1.05, 1.29)
Brisbane	1.01 (0.97, 1.09)	1.00 (0.96, 1.09)	1.01 (0.97, 1.10)	0.96 (0.92, 1.01)	0.95 (0.93, 1.00)	0.99 (0.97, 1.04)	1.08 (1.03, 1.19)	0.96 (0.92, 1.06)	0.93 (0.88, 1.03)
Melbourne	1.00 (0.98, 1.04)	1.01 (0.99, 1.05)	0.96 (0.94, 1.01)	0.99 (0.94, 1.04)	0.98 (0.96, 1.04)	0.96 (0.93, 1.01)	0.96 (0.92, 1.04)	0.90 (0.86, 0.98)	1.01 (0.96, 1.11)
Perth	0.95 (0.92, 1.01)	0.93 (0.90, 1.00)	0.97 (0.93, 1.03)	1.01 (0.95, 1.08)	0.99 (0.95, 1.06)	1.02 (0.98, 1.09)	0.98 (0.90, 1.15)	0.98 (0.90, 1.15)	1.05 (0.97, 1.24)
Sydney	1.06 (1.02, 1.11)	1.04 (1.02, 1.09)	0.99 (0.99, 1.04)	0.98 (0.95, 1.02)	0.96 (0.95, 1.00)	0.97 (0.96, 1.01)	0.94 (0.90, 1.01)	0.87 (0.83, 0.94)	0.99 (0.95, 1.07)
**Canada**									
Alberta	1.03 (1.01, 1.09)	0.95 (0.93, 1.01)	1.00 (0.98, 1.05)	0.99 (0.96, 1.02)	1.29 (1.18, 1.53)	1.24 (1.13, 1.47)	0.74 (0.65, 0.93)	1.12 (0.99, 1.44)	0.67 (0.60, 085)
Halifax	1.04 (0.99, 1.05)	0.95 (0.98, 1.04)	0.99 (0.95, 1.08)	0.98 (0.84, 1.13)	1.12 (1.03, 1.31)	1.09 (1.01, 1.26)	0.83 (0.72, 1.12)	1.11 (0.97, 1.47)	0.91 (0.79, 1.19)
Toronto	1.00 (0.99, 1.02)	0.97 (0.96, 0.99)	1.00 (0.99, 1.02)	0.90 (0.78, 1.05)	1.03 (1.02, 1.07)	0.98 (0.97, 1.02)	0.97 (0.94, 1.03)	1.09 (1.05, 1.16)	1.101 (0.98, 1.08)
Vancouver	0.95 (0.91, 1.01)	1.05 (1.01, 1.11)	1.01 (0.98, 1.08)	1.02 (0.05, 0.92)	0.93 (0.88, 1.02)	0.93 (0.89, 1.03)	1.02 (0.98, 1.11)	1.01 (0.96, 1.10)	1.06 (1.01, 1.15)
**USA**									
Baltimore	1.08 (1.06, 1.16)	1.04 (1.01, 1.11)	1.08 (1.05, 1.15)	0.95 (0.87, 1.04)	0.94 (0.90, 1.02)	0.91 (0.87, 0.98)	1.10 (0.96, 1.45)	1.21 (1.02, 1.67)	0.88 (0.76, 1.20)
Philadelphia	1.05 (1.02, 1.12)	1.00 (0.97, 1.06)	1.02 (0.99, 1.09)	0.99 (0.81, 1.22)	0.99 (0.95, 1.08)	0.90 (0.86, 0.99)	0.74 (0.62, 1.03)	0.86 (0.71, 1.23)	1.16 (0.99, 1.60)
Providence	0.99 (0.92, 1.13)	0.97 (0.90, 1.10)	0.86 (0.80, 0.97)	0.91 (0.85, 0.98)	0.93 (1.02, 1.24)	1.14 (1.04, 1.36)	0.91 (0.70, 1.55)	0.81 (0.60, 1.48)	1.07 (0.80, 1.88)

NOTE: IPD, invasive pneumococcal disease; CI, confidence interval.

Odds ratios generated using conditional logistic regression, adjusted simultaneously for mean temperature, absolute humidity, normalized influenza and ultraviolet index at lags of 1-3 weeks.

© 2020 Berry I et al. *JAMA Network Open.*
